# Asteraceae Paradox: Chemical and Mechanical Protection of *Taraxacum* Pollen

**DOI:** 10.3390/insects11050304

**Published:** 2020-05-14

**Authors:** Maryse Vanderplanck, Hélène Gilles, Denis Nonclercq, Pierre Duez, Pascal Gerbaux

**Affiliations:** 1 Laboratory of Zoology, Research Institute for Biosciences, University of Mons-UMONS, Place du Parc 23, 7000 Mons, Belgium; helgilles@hotmail.com; 2 Laboratory of Histology, Faculty of Medicine and Pharmacy, University of Mons-UMONS, Place du Parc 23, 7000 Mons, Belgium; denis.nonclercq@umons.ac.be; 3 Unit of Therapeutic Chemistry and Pharmacognosy, Faculty of Medicine and Pharmacy, University of Mons-UMONS, Place du Parc 23, 7000 Mons, Belgium; pierre.duez@umons.ac.be; 4 Organic Synthesis and Mass Spectrometry Laboratory, Research Institute for Biosciences, University of Mons-UMONS, Place du Parc 23, 7000 Mons, Belgium; pascal.gerbaux@umons.ac.be

**Keywords:** Asteraceae, pollen defenses, generalist floral visitors, *Bombus terrestris*, chemical protection, mechanical protection

## Abstract

Excessive pollen harvesting by bees can compromise the reproductive success of plants. Plants have therefore evolved different morphological structures and floral cues to narrow the spectrum of pollen feeding visitors. Among “filtering” mechanisms, the chemical and mechanical protection of pollen might shape bee-flower interactions and restrict pollen exploitation to a specific suite of visitors such as observed in Asteraceae. Asteraceae pollen is indeed only occasionally exploited by generalist bee species but plentifully foraged by specialist ones (i.e., Asteraceae paradox). During our bioassays, we observed that micro-colonies of generalist bumblebee (*Bombus terrestris* L.) feeding on *Taraxacum* pollen (Asteraceae) reduced their pollen collection and offspring production. Bees also experienced physiological effects of possible defenses in the form of digestive damage. Overall, our results suggest the existence of an effective chemical defense in Asteraceae pollen, while the hypothesis of a mechanical defense appeared more unlikely. Pre- and post-ingestive effects of such chemical defenses (i.e., nutrient deficit or presence of toxic compounds), as well as their role in the shaping of bee-flower interactions, are discussed. Our results strongly suggest that pollen chemical traits may act as drivers of plant selection by bees and partly explain why Asteraceae pollen is rare in generalist bee diets.

## 1. Introduction

Through pollen collection, bees act concurrently as effective pollinators and herbivores, since both larvae and adults feed exclusively on pollen and nectar [1,2]. Like other herbivorous insects, bees display a high diversity of interactions with their host plants, from strict specialization (i.e., oligolecty, where bees collect pollen from flowers of a single genus, subfamily, or family) to broad generalization (i.e., polylecty, where bees exploit flowers of more than one plant family) [3,4,5]. Regardless of floral specialization, bees display numerous adaptations to discriminate among plant species and to enhance floral resource foraging [1,6,7,8], which can compromise the reproductive success of plants. For instance, bees can remove 95.5% of the pollen produced by flowers of *Campanula rapunculus* (Campanulaceae) [9] and some solitary species require the entire content of more than 30 flowers, even more than 1000 flowers, to feed a single larva [7]. In response to excessive pollen harvesting, flowering plants have drawn up complex defense mechanisms and adaptations. In fact, flowers have evolved several morphological traits that can be viewed as adaptations preventing excessive pollen harvesting by bees, such as heteranthery, nototribic flowers (i.e., dorsal anthers hidden in the upper lip of the flower, such as in Lamiaceae), keel flowers (i.e., ventral anthers hidden in a boat-shaped keel formed by the fusion of the two lower petals of the flower, such as in Fabaceae), floral tubes, poricidal anthers and progressive pollen release (reviewed in [8]).

Selection may also act on pollen traits to narrow the spectrum of pollen feeding visitors. For instance, although Asteraceae are ubiquitous in most temperate habitats [10], they are only occasionally exploited by polylectic species such as *Bombus* [11,12] and *Colletes* [4] (i.e., Asteraceae paradox [4]). This Asteraceae avoidance cannot be explained by complex floral morphology, since compound inflorescences ensure an easy access to both pollen and nectar over an extended time period [4]. The failure of several unspecialized bee species to develop on Asteraceae pollen rather suggests that it may possess unfavorable or protective properties so that bees might require physiological adaptations to use it [13,14,15,16,17,18,19]. Although Asteraceae pollens are known to have low protein content, this is seemingly not the only reason for the inadequacy of their pollen [20]. The pollen may actually lack other essential nutrients, contain toxins, or display a low digestibility [4,13,17,20,21,22]. Such pollen protections probably shape bee–flower interactions to lead to a narrowing of the spectrum of pollen-feeding visitors in Asteraceae.

It is largely assumed that the synthesis of secondary metabolites constitutes a complex system of chemical defenses in plants against herbivorous insect attacks [23,24]. Although these chemical compounds are mainly studied in vegetative parts, some obviously occur in nectar and pollen of flowering plants, with bee pollinators then exposed to their biological activities [24,25,26,27,28,29,30,31,32]. For instance, sesquiterpene lactones are characteristic secondary metabolites in Asteraceae [33], with high chemotaxonomic specificity [34]. Although they probably have evolved as defense through their deterrence to herbivores [35], they also occur in pollen [36] and may display insecticidal activities [37]. Among chemical defenses, constraints could also act through nutrient availability. Although proteins are often regarded as a reference in terms of nutritional quality, lipids are also important [38,39,40,41], including sterols, essential compounds in bee physiology (e.g., pupation, ovary development) that are exclusively exogenous [42,43]. The fact that ∂7-sterols often occur in Asteraceae pollen in higher proportions than more common and usable sterols (i.e., 24-methylenecholesterol, ß-sitosterol, and ∂5-avenasterol) may indicate a defense mechanism against excessive pollen harvesting [22]. In addition to these variations of pollen primary and secondary metabolites, pollen also varies in its wall resistance properties, which may result in incomplete digestion. Transmission electron microscopy has revealed that Asteraceae pollen possesses a thick multilayer wall [44], which might inhibit the extraction of nutrients and act as a mechanical defense [17,21]. Despite these hypotheses, it is unclear why Asteraceae pollen is unsuitable for most bee species and the Asteraceae paradox remains unsolved. In the present study, bumblebee micro-colonies (*Bombus terrestris* L.) were forced to feed on different diets to investigate the unfavorable properties of *Taraxacum* pollen. From the observed patterns in foraging behavior, larval development, and digestive damages, we infer possible mechanical or chemical protective properties of Asteraceae pollen.

## 2. Material and Methods 

### 2.1. Model System

*Taraxacum officinale* Weber (Asteraceae), or common dandelion, is an apomictic perennial herbaceous species that originated in Eurasia, although is now cosmopolitan. Indeed, this invasive species is capable of establishing under diverse environmental conditions and is now globally distributed [45]. This weed has attractive floral capitula and it is frequently observed among native vegetation [46,47], attracting a wide variety of insect pollinators including Hymenoptera, Diptera, and Lepidoptera [48]. Such attractiveness can be explained by the fact that each floret produces abundant quantities of pollen and nectar that are easily accessible [49]. Dandelion is in flower for most of the year but has a peak flowering time from late March to May when many pollinators emerge after winter, including the buff-tailed bumblebee queens (*Bombus terrestris* L.). Dandelion flowers may then be a useful food resource to early pollinators (i.e., emerging in early spring) when other flower species are often few and sparse. Because this species is so widespread and can be locally abundant, it is important to investigate whether its pollen might have negative effects on generalist pollinators and determine its protective properties.

*Bombus terrestris* is one of the most abundant and widespread bumblebee species in the west Paleartic. This social species is a highly polylectic bumblebee foraging on hundreds of different plant species and numerous plant families [12,50,51]. As a consequence, it plays a relevant role as a pollinator in wild and cultivated plant communities [50]. However, colonies do not show equivalent development on all pollen species [22] and only occasionally exploit Asteraceae pollen [11,12].

### 2.2. Pollen Diets

**Figure 1:**
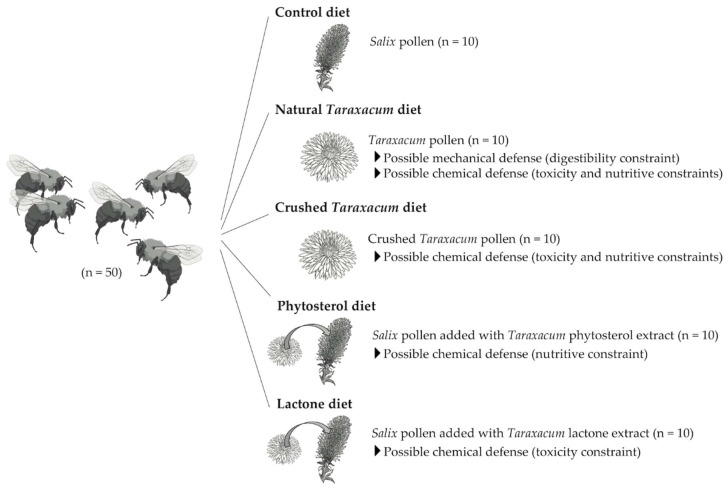
Experimental design and summary of diet treatments provided to *B. terrestris*. Each micro-colony consisted of 5 workers fed for 35 days. Mortality, offspring production, and resource collection (i.e., pollen and syrup) were monitored during or at the end of the bioassays.

How structural and chemical properties of *Taraxacum* pollen can impact pollinator behavior, performance, and health was investigated by the use of five different pollen treatments: (i) control diet, (ii) natural *Taraxacum* diet, (iii) crushed *Taraxacum* diet, (iv) phytosterols added to control diet, and (v) lactones added to control diet (Figure 1). The control diet consisted of pollen loads with a dominance of *Salix* sp. mixed with inverted sugar syrup (BIOGLUC^®^, Biobest, Westerlo, Belgium) to obtain consistent candies. *Salix* pollen is described as an excellent resource for *B. terrestris* colony development and is then unlikely to display traits acting as defenses [20,22,52]. The natural *Taraxacum* diet consisted of pollen loads with a dominance of *Taraxacum* sp. mixed with inverted sugar syrup to obtain candies. This pollen diet retained both mechanical and chemical properties of Asteraceae pollen that could act as defenses against generalist bees. The crushed *Taraxacum* diet consisted of *Taraxacum* pollen crushed under liquid nitrogen and mixed with inverted sugar syrup to obtain candies. Microscopical examination revealed that at least 70% of *Taraxacum* pollen grains were broken after crushing. This treatment allows for eliminating the mechanical protection of *Taraxacum* pollen while retaining the chemicals that could act as defenses. The phytosterol- and lactone-supplemented diets contained chemicals extracted from *Taraxacum* pollen (Supplement S1) mixed with the control diet, which eliminated eventual mechanical protection of *Taraxacum* pollen but conserved either nutrients or eventual defense secondary metabolites. The chemical extracts of *Taraxacum* pollen were mixed with the control diet in ratios that mimic their proportions in the *Taraxacum* pollen diet. All treatment diets contained the extract solvent (aqueous ethanol, 1:1; 0.4 mL/g of diet) to control for its potential negative effects when assessing the added chemical treatments (i.e., phytosterol and lactone diets). Pollen loads of *Salix* and *Taraxacum* were purchased from the company “Ruchers de Lorraine” and were sold as organic nutrition complement (i.e., free of pesticides). 

### 2.3. Bioassays

#### 2.3.1. Experimental Design

The experiments were conducted at the University of Mons from February to May 2016. Two-day-old workers of *Bombus terrestris* were collected from five different colonies (i.e., A, B, C, D, and E) provided by Biobest *bvba* (Westerlo, Belgium). They were divided into 50 micro-colonies (i.e., 10 micro-colonies per colony) of 5 workers and placed in different plastic boxes (10 × 16 × 16 cm). The micro-colonies were distributed among the different diets to ensure homogeneity of origins (i.e., 2 micro-colonies from each founding colony per treatment for a total of 10 micro-colonies per treatment) (Figure 1). These micro-colonies were fed ad libitum with sugar syrup (BIOGLUC^®^, Biobest) and pollen candies in a dark room at 27 °C and 76% relative humidity during the 35-day period following the first episode of egg-laying of a worker. New pollen candies were provided every 2 days (0.5, 1.0, or 1.5 g depending on the age of the micro-colony) to avoid nutrient alteration. Syrup and pollen supplies as well as monitoring of micro-colonies were conducted in a darkroom under red light in order to avoid disturbing colonies, as bees do not detect this range of the light spectrum. Such a method using queenless *Bombus terrestris* micro-colonies for testing the suitability of pollen diets has been validated previously and is accepted as a good estimate of queen-right colony development [53]. 

#### 2.3.2. Micro-Colony Performance

Feeding response and micro-colony development were evaluated based on: (i) composition (i.e., number of eggs, non-isolated larvae, isolated larvae, pupae, non-emerged and emerged drones) and fresh weight of offspring, (ii) larval ejection, (iii) pollen collection (i.e., amount of pollen consumed and stored) (fresh matter), (iv) pollen efficiency (i.e., the weight of hatched offspring divided by the total pollen collected per micro-colony), and (v) syrup collection (i.e., amount of syrup consumed and stored) (parameters adapted from [53]). Pollen and syrup collections were measured by weighing pollen candies and syrup container before their introduction into the micro-colony and after their removal (i.e., every 2 days). All weight parameters (i.e., brood weight, pollen collection, and syrup collection) were standardized by the total weight of workers in the micro-colonies to avoid potential bias from worker activities (i.e., consumption and brood care). 

#### 2.3.3. Digestive Damages

For each treatment, a total of five bumblebee workers were randomly collected among the different micro-colonies (i.e., one worker per founding colony) and cold-anesthetized prior to the cutting of the abdomen. The abdomen cuticle was slightly incised and a lateral part was removed to facilitate the fixation, dehydration, and paraffin-embedding processes. The prepared abdomens were fixed by immersion in Duboscq–Brazil fluid (composition: formalin/acetic acid/ethanol containing 1% picric acid/distilled water, respective proportions: 260/70/425/245, v/v) for 48 h. After dehydration and paraffin-embedding, abdomens were cut in serial sections of 5 µm thicknesses on a Reichert-Jung^®^ 2040 microtome with the use of a softening agent (Mollifex^TM^) and placed on silane-coated glass slides. After rehydration, the sections were stained with hematoxylin, Ponceau-acid Fuchsin-Orange G and light green (Masson’s Trichrome stain) to allow histological examination. Tissue sections were examined and photographed with the help of a research optical microscope (Leitz^®^ Orthoplan, Leica, Wetzlar, Germany) equipped with a high sensibility camera (Leica^®^ DFT7000 T, Wetzlar, Germany) using the TWAIN driver and Corel PHOTO-PAINT^®^ software.

### 2.4. Data Analysis

To test for differences in syrup collection, fitness (offspring mass; drone mass; number of individuals within each developmental stage), pollen efficiency, and larval ejection among diet treatments, we fitted a general linear mixed effects models with diet treatment as a fixed effect and colony as a random factor. Syrup collection, total offspring mass, drone mass, and pollen efficiency per micro-colony were analyzed using models with a Gaussian error structure (i.e., normally distributed residuals, “lme” function, R-package “nlme”; [54]). For drone mass, we added micro-colonies as a hierarchical random effect in the model so that the non-independence of the data could be taken into account (i.e., several data points per micro-colony). We also assessed pollen collection over time with the day of experiment as a continuous fixed effect and micro-colonies as a hierarchical random effect in the model (repeated measures). As normality of the residuals was not respected, we used a gamma-distribution model and a logit link function, which is adapted for continuous and non-normal data. Larval ejection was analyzed using a binomial model with the number of ejected larvae and the total number of living offspring produced per micro-colony as a bivariate response (“glmer” function, R-package “lmerTest”; [54]), with an observation-level random effect added to the model to account for overdispersion (i.e., each data point received a unique level of random effect that modelled the extra-parametric variation present in the data; [55]). Numbers of individuals within each developmental stage per colony were assessed using models with a Poisson distribution for count data after checking for overdispersion (“glmer” function, R-package “lmerTest”; [54]). An observation-level random effect was added to the Poisson models when data overdispersion occurred [55]. When a significant effect was found (*p* < 0.01), multiple pairwise comparison tests were performed using Tukey contrasts and False Discovery Rate adjustment to determine how diet treatments significantly differed from each other (“glht” function, R-package “multcomp”; [56]). All analyses were performed in R version 3.4.0 [57].

## 3. Results

### 3.1. Micro-Colony Performance

#### 3.1.1. Resource Collection

**Figure 2:**
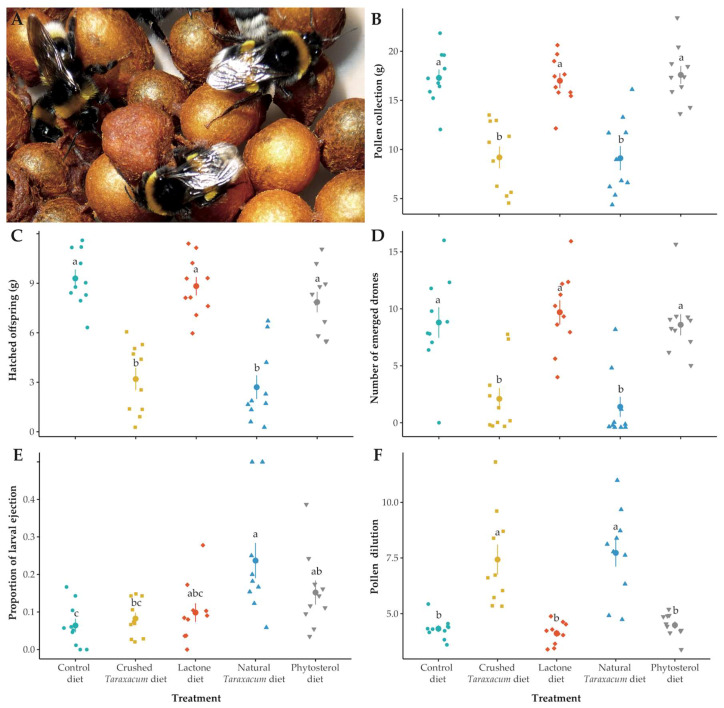
Effects of diet treatments on *B. terrestris* in micro-colonies. (**A**) Details of a micro-colony, photography N. Roger, (**B**) pollen collection in each micro-colony across treatments, (**C**) pollen dilution in each micro-colony across treatments, (**D**) total mass of hatched offspring in each micro-colony across treatments, (**E**) proportion of ejected larvae in each micro-colony across treatments, (**F**) number of emerged drones in each micro-colony across treatments. Each small data point represents a micro-colony and large points are mean values of each treatment. Error bars indicate the standard error of means. Letters indicate significance at *p* < 0.01.

We found a significant effect of day (χ^2^ = 366.60, df = 1, *p* < 0.001) and diet treatment (χ^2^ = 53.59, df = 4, *p* < 0.001) on pollen collection by *B. terrestris* micro-colonies. Post-hoc analyses indicated that pollen consumption increased over time for all diet treatments (Figure S1) but that micro-colonies fed natural and crushed *Taraxacum* diets collected half as much pollen as micro-colonies fed other diets (Figure 2B, Table S1). We also found a significant effect of diet treatment on syrup collection (χ^2^ = 18.73, df = 4, *p* < 0.001). Post-hoc test showed that micro-colonies fed the phytosterol diet collected significantly higher amounts of syrup than micro-colonies fed natural and crushed *Taraxacum* diets. Micro-colonies fed the control diet did not differ from micro-colonies fed the phytosterol and natural *Taraxacum* diets, while micro-colonies fed lactone diet did not differ from micro-colonies fed all other treatments (Table S1). 

#### 3.1.2. Fitness

We found a significant effect of diet treatment on the total mass of hatched offspring (i.e., all developmental stages except eggs) produced by *B. terrestris* micro-colonies (χ^2^ = 122.41, df = 4, *p* < 0.001). Post-hoc analyses indicated that micro-colonies fed natural and crushed *Taraxacum* diets exhibited significantly lower production of hatched offspring than micro-colonies fed all other treatments (Figure 2C; Table S1). 

While all micro-colonies produced eggs, we found a significant effect of treatment on the number of non-isolated larvae produced (χ^2^ = 15.54, df = 4, *p* = 0.004), with post-hoc Tukey analyses showing that micro-colonies fed the natural *Taraxacum* diet produced less non-isolated larvae than micro-colonies fed the control diet (Table S1). We also found a significant effect of treatment on the number of pupae produced (χ^2^ = 32.50, df = 4, *p* < 0.001), with micro-colonies fed the control and phytosterol diets producing more pupae than bees in the natural and crushed *Taraxacum* treatments Table S1). We then assessed if the diet treatment also affected the ability of a micro-colony rearing their offspring to adulthood and found a significant difference in the number of emerged drones (χ^2^ = 49.88, df = 4, *p* < 0.001), while their mass did not differ (χ^2^ = 10.45, df = 4, *p* = 0.033). Post-hoc pairwise comparisons showed that micro-colonies fed control, phytosterol, and lactone treatments produced more adult offspring than micro-colonies fed natural and crushed *Taraxacum* diets, which produced adults only occasionally (Figure 2D; Table S1). We found no significant effects of treatment either on the number of isolated larvae (pre- and post-defecating stages) or on the number of non-emerged drones (Table S1).

#### 3.1.3. Stress Response

In response to a diet stress, adult bumblebees may display peculiar behavior, such as larval ejection from the brood [20] or pollen dilution [22]. We found a significant effect of diet treatment on the proportion of ejected larvae in micro-colonies (χ^2^ = 21.14, df = 4, *p* < 0.001). A post-hoc Tukey test showed that micro-colonies fed the natural *Taraxacum* and phytosterol treatments exhibited higher larval ejection than micro-colonies fed the control treatment. Larval ejection in micro-colonies fed the lactone diet did not differ from all other diets, while larval ejection in micro-colonies fed the crushed *Taraxacum* treatment was significantly lower than in micro-colonies fed the natural *Taraxacum* treatment, but did not differ from micro-colonies fed the control, lactone, and phytosterol diets (Figure 2E; Table S1). We also found a significant effect of diet treatment on pollen dilution (χ^2^ = 95.52, df = 4, *p* < 0.001). Post-hoc analyses indicated that bumblebees fed natural or crushed diets collected a significantly higher amount of syrup per gram of pollen compared to the other diets (Figure 2F; Table S1).

Another evaluated stress response was pollen diet efficiency, which highlights when a micro-colony needs to consume more pollen to produce offspring, and, in turn, could be indicative of a digestibility constraint or nutrient deficiency. We found a significant effect of treatment on pollen diet efficiency (χ^2^ = 100.54, df = 4, *p* < 0.001), with micro-colonies fed the natural and crushed *Taraxacum* treatments having a lower pollen efficiency than bees in all other treatments (Table S1).

### 3.2. Digestive Damage

#### 3.2.1. General Histology

**Figure 3:**
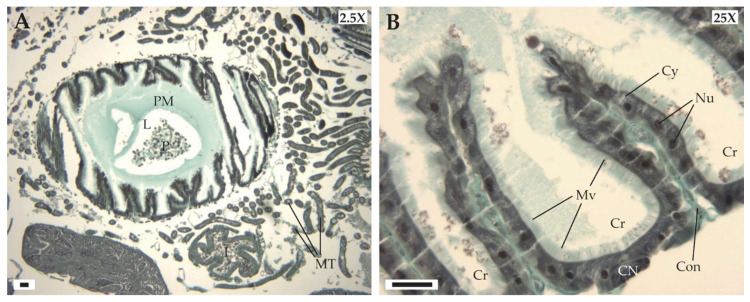
(**A**) Cross-section of the digestive tract of bumblebee worker. (**B**) Details of intestinal crypts and mesenteric epithelium. CN: cellular nidi; Con: connective tissue; Cr: intestinal crypts; Cy: granular cytoplasm; I: ileum; L: lumen; MT: Malpighian tubules; Mv: microvilli; P: pollen grains; PM: peritrophic membrane. Scale bar, 50 μm.

The bumblebee digestive tract, as in all arthropods, is composed of a cuticle-lined foregut (stomodaeum), a midgut (mesenteron), and a cuticle-lined hindgut (proctodaeum). The mesenteron is the principal site of nutrient digestion and absorption, as well as the first line of defense against the absorption of ingested plant allelochemicals (Figure 3A). As such, it contains high activities of digestive enzymes, as well as detoxification and antioxidant enzymes. The mesenteron is lined by a laminar structure, the peritrophic membrane (PM), that consists of a network of chitin microfibrils within a matrix of carbohydrates and proteins. This membrane plays a protective role against mechanical damage from abrasive food particles, but also against ingested pathogens and certain plant allelochemicals while allowing absorption of the nutrients straight into the hemolymph. The peritrophic membrane also compartmentalizes the mesenteron lumen (L) into two compartments, the endoperitrophic space (inside the membrane) that may contain pollen grains (P), and the ectoperitrophic space (outside the membrane). Next along the digestive tract are the Malpighian tubules (MT) that occur in the region of the sphincter (pylorus) separating the mesenteron from the ileum (I). These extensions of the digestive tract are excretory organs that float freely in the bee body cavity. 

The midgut epithelium represents an important interface between the insect and its environment (Figure 3B). It consists of discrete crypts (Cr) and lies on the connective tissue (Con). This epithelium is formed by a single layer of three cell types: (i) columnar or digestive cells responsible for enzyme production and nutrient absorption, (ii) regenerative cells to replace dead cells, and (iii) endocrine cells to secrete peptide hormones. Regenerative cells form cellular nidi (CN) in the base of the intestinal crypts, where cell divisions occur. The columnar cells are the major cell type with numerous microvilli (Mv) forming, at the apical pole, a brush-like border that increases the surface area for both absorption and secretion. They display a slightly granular cytoplasm and, at their center, a large ovoid and euchromatic nucleus (Nu).

#### 3.2.2. Treatment Effects

**Figure 4:**
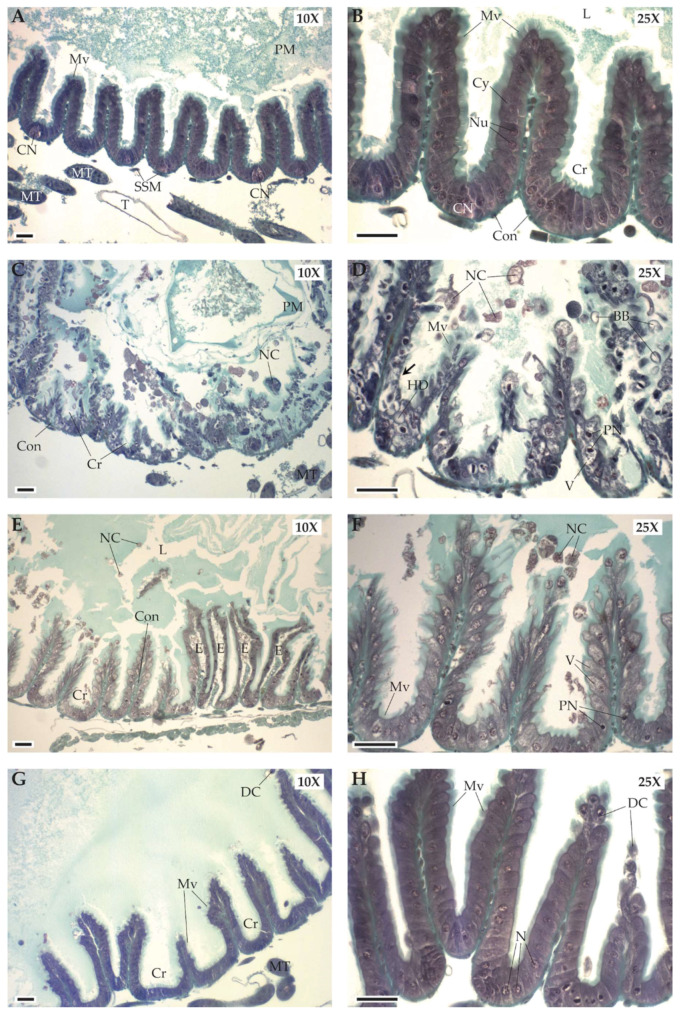
Digestive tracts of the bumblebee workers exposed to the natural *Taraxacum* treatment (**A**,**B**), the crushed *Taraxacum* treatment (**C**,**D**), the phystosterol treatment (**E**,**F**), and the lactone treatment (**G**,**H**). BB: cytoplasmic blebbing; E: interstitial edema; CN: cellular nidi; Con: connective tissue; Cr: intestinal crypt; Cy: granular cytoplasm of digestive cell; DC: detached cells; HD: hydropic degeneration; L: lumen; MT: Malpighian tubules, Mv: microvilli; NC: necrotic cells; Nu: nucleus; PN: pyknotic nuclei; SSM: striated skeletal muscle; T: tracheal system; V: cytoplasmic vacuolization. The arrow indicates the loss of the brush-like border. Scale bar, 50 μm.

The control and natural *Taraxacum* treatments did not cause damage in the digestive tract (Figure 4A,B, Table 1). The mesenteric epithelium displayed a normal organization for both treatments. The morphology of digestive cells appeared to be normal without cytoplasmic vacuolization or pyknotic nucleus. The nuclei had a smooth and regular appearance. No necrotic cells were observed both in the base and at the apex of the intestinal crypts (Cr), which remained well shaped. Cellular nidi (CN) of regenerative cells were observed in the base of the intestinal crypts. Microvilli (Mv) at the apex of the digestive cells were well-developed, without any partial degradation. 

The crushed *Taraxacum* treatment induced marked histopathological alterations in the digestive tract with features of apoptosis and necrosis (Figure 4C,D, Table 1). The intestinal crypts were still visible but several necrotic cells (NC) detached from the epithelium and formed large clusters in the mesenteron lumen. These necrotic cells were observed both in the base and at the apex of the intestinal crypts. Cytoplasmic vacuolization (V) and pyknotic nuclei (PN) were well marked. Disorganization or loss of the brush-like border and hydropic degeneration (HD), as well as cytoplasmic blebbing (BB) were also frequently observed. 

The digestive tract damage of bees exposed to the phytosterol treatment were less severe (Figure 4E,F, Table 1). The intestinal crypts were still well organized. However, isolated necrotic cells (NC) and cellular debris were observed in the mesenteron lumen. Interstitial edema (E) in the connective tissue that forms the central axes of intestinal crypts was also observed. Occurrence of pyknotic nuclei (PN) was lower than for crushed *Taraxacum* treatment and we did not observe hydropic degeneration despite cytoplamic vacuolization (V). The brush-like border was still homogeneous with well-developed microvilli (Mv).

No degeneration of epithelial cells was observed in the digestive tract of bees exposed to the lactone treatment (Figure 4G,H, Table 1). The mesenteron displayed a normal morphology as in the control treatment: the intestinal crypts were well-formed with a homogeneous brush-like border. Only some cells detached at the apex of the intestinal crypts (DC), which was probably due to normal cell renewal.

## 4. Discussion

Our study assessed the impact of potential mechanical and chemical pollen defenses of Asteraceae on the fitness of a generalist pollen forager through a mechanistic lab experiment. Bumblebee micro-colonies fed non-*Taraxacum* pollen increased their pollen collection over time twice as much as the micro-colonies fed natural and crushed *Taraxacum* pollen. Micro-colonies fed natural and crushed *Taraxacum* pollen produced less offspring and especially reared less offspring to adulthood compared with all other treatments. More larvae were ejected from micro-colonies fed natural *Taraxacum* and phytosterol treatments than in the control treatment. Finally, bees in the crushed *Taraxacum* and phytosterol treatments were more likely to exhibit digestive damage than bees fed all other treatments. These results suggest physiological costs associated with the collection of *Taraxacum* pollen.

Our results provide evidence that *Taraxacum* pollen displays defenses that impose severe fitness effects of reduced reproduction to the bumblebee micro-colonies by partly preventing larvae from completing their development to adulthood in both natural and crushed *Taraxacum* treatments. Such low suitability of *Taraxacum* pollen has already been highlighted in previous studies with a diet of pure dandelion pollen that has been reported to impede larval development in non-specialist solitary bee species like mason bees [4,17,18,58], prevent brood production in honey bees [59], and cause 100% larval ejection in bumblebees [15]. The development failure of honeybees on *Taraxacum* pollen [58] has been attributed to its lack in tryptophan and phenylalanine [60] and its deficiency in arginine [61] (i.e., essential amino acids). If this nutritional hypothesis prevails as the sole explanation for pollen defense in *Taraxacum*, our experimental design should have allowed bumblebees micro-colonies to increase their food intake to compensate for any nutrient deficit. However micro-colonies fed natural and crushed *Taraxacum* pollen treatments displayed reduced pollen collection compared with micro-colonies fed all other diet treatments. Such a difference in pollen collection behavior and reproduction reveals that no food compensation has occurred to balance the low efficiency of *Taraxacum* pollen, suggesting that a nutrient deficit cannot be considered the sole pollen defense. However our experiment does not provide support for totally rejecting the nutritional hypothesis as one of the effective defense mechanisms in Asteraceae pollen.

The reduced pollen collection on *Taraxacum* diets may be indicative of feeding deterrence that could be due to low digestibility (i.e., physical defense), nutrient deficit, or presence of toxic compounds (i.e., chemical defense), which may be either directly toxic or interfere with nutrient assimilation [62,63]. Regarding the hypothesis of physical defense, Asteraceae pollen grains possess a thick multilayer wall that retains sporopollenin [44], as well as high amounts of pollenkit [16]. These structural properties of pollen grains could interfere with the nutrient assimilation process and render digestion difficult, requiring specific enzymes for extracting nutrients [17,21]. Such a digestibility constraint has been reported for honeybees fed *Taraxacum* [64] and could be responsible for the failure of *Chelostoma rapunculi* (oligolectic on *Campanula* genus), *C. florisomne* (oligolectic on *Ranunculus* genus), and *Hoplitis adunca* (oligolectic on *Echium* genus) to develop on Asteraceae pollen [17]. However, the fact that pollen efficiency in micro-colonies fed crushed *Taraxacum* treatment was close to that in micro-colonies fed natural *Taraxacum* treatment indicates that *Taraxacum* unsuitability for bumblebees is not due to complex pollen structure (i.e., mechanical defense) but rather to either missing essential dietary components or presence of toxic compounds that interfere with physiological processes (i.e., chemical defense). 

Chemical defenses of pollen may actually result in both pre-ingestive effects, by reducing pollen collection, and post-ingestive effects, by reducing reproduction [65]. Pre-ingestive effects were evident in our study since micro-colonies fed natural and crushed *Taraxacum* pollen treatments displayed reduced pollen collection. Such regulation of food intake can be explained by the ability of bumblebees to continuously assess the pollen quality through chemotactile cues (nutritional and non-nutritional cues) prior to ingestion [66,67]. While it has been demonstrated that individual bumblebee foragers are able to assess the overall amino acid (i.e., free and proteinogenic) and lipid contents of pollen to provide their colonies with highly suitable food [41,67], toxic substances may also be detected and subsequently affect foraging decision (i.e., chemosensory perception translated into behavioral response). For instance, mitigation of unsuitable chemical properties may be achieved through pollen mixing behavior during foraging (i.e., “toxin” dilution; [68]). The fact that bumblebees feeding on *Taraxacum* pollen (i.e., natural and crushed treatments) collected a higher quantity of syrup, which corresponds to some kind of mixing behavior assumed to dilute the chemicals [22], supports the hypothesis of chemical defense in *Taraxacum* pollen. However our pattern of pollen collection suggests that *Taraxacum* phytosterols and lactones are unlikely to deter bumblebee feeding.

Post-digestive effects were less obvious than pre-ingestive ones in our experiment since reduced reproduction in micro-colonies fed natural and crushed *Taraxacum* pollen treatments might be due to either effective chemical defense of ingested pollen or malnutrition linked to the reduced pollen collection. Although our data do not allow one hypothesis to prevail over another, digestive damage in bumblebees fed the crushed *Taraxacum* diet might be indicative of an effective chemical defense afforded by toxic compounds in the pollen. In crushing the *Taraxacum* pollen, we may actually have increased the level of chemical defenses by improving the bioavailability of toxins but also the level of physical damage by creating smaller shards of exine, which are more abrasive for the digestive tract than intact exine; these, however, do not account for an original mechanical defense as these shards were produced by crushing the *Taraxacum* pollen (i.e., experimental artifact) [65]. Nevertheless, the chemical hypothesis is supported by our finding that bees fed the phytosterol diet also displayed digestive damage. As in most Asteraceae species, *Taraxacum* pollen displays a high proportion of δ7-sterols. Such an occurrence of these quite rare phytosterols may be foreseen as a defense mechanism against excessive pollen harvesting and could have contributed to reduced reproduction in natural and crushed *Taraxacum* treatments. The peculiar phytosterolic composition of Asteraceae pollen has been already pinpointed as detrimental to bumblebee micro-colony development (i.e., slowing down of micro-colony development and increase of larval mortality) [22,69]. Such a function of δ7-sterols as a post-ingestive defense against herbivory has also been suggested in other insect groups, such as grasshoppers [70,71,72,73] and two lepideptoran species [74,75]. By contrast, our results suggest no negative post-ingestive effects of lactone treatment on micro-colony reproduction nor worker digestive tracts. It is important to note that the impacts of pollen diet on the bee gut microbiota should also be taken into consideration when investigating pollen post-ingestive defenses. Bacterial gut symbionts have been shown to be important for parasite resistance and degradation of secondary plant metabolites, as well as for pollen digestion and nutrient assimilation [76,77,78,79,80]. The bee microbiome is, therefore, a crucial factor affecting bee health. It is already known that nutrient content can modulate the bacterial composition in the bumblebee gut [81]. In the same way, secondary compounds in pollen could affect bees via changes in the gut microbiota, acting as post-ingestive defenses.

Overall, the chemical traits of *Taraxacum* pollen acting as defenses against excessive harvesting merit further investigation. On one hand, many other secondary compounds could account for the chemical defense of *Taraxacum* pollen (e.g., alkaloids). On the other hand, nutrient concentration and composition may also act as chemical defense since both excessive and deficient amounts of macronutrients are detrimental [82]. For instance, too high concentration of proteins or lipids may affect survival and reproduction [41,83,84], and malnutrition can also reduce investment in producing offspring [20]. The nutrient balance is therefore highly delicate and should not be neglected when investigating pollen defense mechanisms.

## 5. Conclusions

The observed patterns clearly point to the importance of chemical and mechanical defenses in shaping the relationships between bees and flowers, as highlighted in traditional plant–herbivore interactions [85]. We found that the mechanical defenses of Asteraceae pollen appear quite negligible, while chemical protection may act through the presence of toxic compounds or imbalanced nutrient content. Such unsuitable chemical properties of pollen can be assessed by bees based on chemotactile cues and induce a feeding deterrence resulting in reduced pollen collection (i.e., pre-ingestive effects). Malnutrition, as well as physiological costs linked to detoxification, can also reduce offspring production or increase mortality in bees (i.e., post-ingestive effects). These pre- and post-ingestive effects may explain why Asteraceae pollen is rarely a component of the diet of bumblebees, including the most polylectic species (e.g., *Bombus terrestris*) [12], despite the diversity and the abundance of this plant family [10]. However, it is important to note that, compared with post-ingestive defenses, pre-ingestive defenses may benefit both partners as they are less costly for plants (i.e., reduction of pollen lost) and allow bees to avoid physiological damage (i.e., reduced reproduction and increased mortality or digestive damage). Future experiments should aim to accurately determine the pollen traits responsible for chemical defense in Asteraceae pollen and help to elucidate the Asteraceae paradox in bee–plant interactions, considering host–microbiome interactions.

## Figures and Tables

**Table 1:** Signs of histopathological alterations in the digestive tract of *B. terrestris* workers fed various diet treatments.

Digestive Damages	Treatment				
	Control	Natural *Taraxacum*	Crushed *Taraxacum*	Phytosterol	Lactone
Cytoplasmic blebbing			X		
Cytoplasm vacuolization			X	X	
Disorganization of the brush-like border			X		
Hydropic degeneration			X		
Interstitial edema				X	
Necrotic cells			X	X	
Pyknotic nuclei			X	X	

## Supplementary Materials

The following are available online at https://www.mdpi.com/2075-4450/11/5/304/s1, Supplement S1: Chemical extraction of Taraxacum pollen. Table S1: Effects of diet treatments on B. terrestris in micro-colonies. Figure S1: Pollen collection by B. *terrestris* in micro-colonies over time.
