# Supplementary material for: Asteraceae Paradox: Chemical and Mechanical Protection of Taraxacum Pollen
Source: Insects. 2020 May 14;11(5):304. doi: 10.3390/insects11050304 (PMC7290674; doi:10.3390/insects11050304)
Supplement: Supplementary file 1 [file insects-11-00304-s001.zip › insects-798731-supplementary.docx]

Supplementary materials

Asteraceae paradox: chemical and mechanical protection of *Taraxacum* pollen

Maryse Vanderplanck ^1,*^, Hélène Gilles ^1^, Denis Nonclercq ^2^, Pierre Duez ^3^ and Pascal Gerbaux ^4^

^1^ Laboratory of Zoology, Research Institute for Biosciences, University of Mons - UMONS, Place du Parc 23, 7000 Mons, Belgium; maryse.vanderplanck@umons.ac.be

^2^ Laboratory of Histology, Faculty of Medicine and Pharmacy, University of Mons - UMONS, Place du Parc 23, 7000 Mons, Belgium; denis.nonclercq@umons.ac.be

^3^ Unit of Therapeutic Chemistry and Pharmacognosy, Faculty of Medicine and Pharmacy, University of Mons - UMONS, Place du Parc 23, 7000 Mons, Belgium; pierre.duez@umons.ac.be

^4^ Organic Synthesis and Mass Spectrometry Laboratory, Research Institute for Biosciences, University of Mons - UMONS, Place du Parc 23, 7000 Mons, Belgium; pascal.gerbaux@umons.ac.be

***** Correspondence: [maryse.vanderplanck@umons.ac.be](mailto:maryse.vanderplanck@umons.ac.be); Tel.: +32 65 37 34 36

Supplement S1 – Chemical extraction of *Taraxacum* pollen

*Phytosterols –* The *Taraxacum* phytosterol extract was obtained by Soxhlet-extraction of the *Taraxacum* pollen into petroleum ether 40-60°C for 24h (protocol adapted from [1]). The petroleum ether was then evaporated at 60°C under reduced pressure and the chemical extract was resuspended in a 2 M KOH methanolic solution. The dried extract was saponified for 1 h at 80°C, diluted with distilled water after cooling and extracted three times against diethyl ether. The pooled ether extracts were washed three times with distilled water and dried over anhydric sodium sulfate. The diethyl ether was evaporated under reduced pressure at 35 °C to dryness. The extract was then resuspended in acetonitrile to undergo SPE purification with consecutive elution by water, acetonitrile and acetonitrile : toluene (3:1, v/v). The final phytosterol extract was evaporated and resuspended in 100 mL of an aqueous ethanolic solution (50:50, v/v). The phytoterolic extract was characterized by gas chromatography coupled to a flame ionization detector (GC-FID).

The GC-FID analysis was performed on a Thermo Trace GC Ultra gas chromatograph equipped with a flame ionization detector, an AS 3000 autosampler and a HP-5 column (5%-phenyl-methylpolysiloxane stationary phase; 30 m column length; 0.25 mm inner diameter; 0.25 mm film thickness; Machery-Nagel). The initial temperature of the column was maintained 1 min at 60 °C, then programmed to 290 °C at 30 °C min-1 and maintained for 12.5 min. After this isotherm at 290 °C, the temperature was increased to 320 °C (30 °C min-1) then isothermal at 320 °C for 3 min. The carrier gas was helium at 1 ml/min. Injection volume was 1 μL in splitless mode (splitless time: 0.80 min). The temperature of the injector was fixed at 300 °C. Detection was performed with 300 Hz FID detector at 330 °C. The flame composition of the detector was: 350 ml/min air, 35 ml/min hydrogen, 30 ml/min nitrogen (makeup gas).

*Taraxacum* pollen diplays phytosterols commonly found in pollen, namely 24-metylenecholesterol, sitosterol and δ5-avenasterol ; but also high abundance of quite rare phytosterols, namely δ7-stigmasterol and δ7-avenasterol (Fig.1). This phytosterolic composition has been already highlighted in *Cirsium* pollen (Asteraceae) [2].


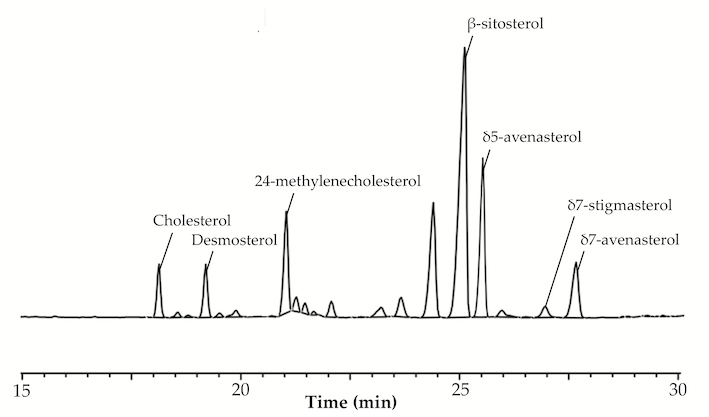


**Figure 1.** GC-FID chromatogram of the sterols of *Taraxacum* pollen loads.

*Lactones –*The *Taraxacum* lactone extract was obtained by Soxhlet-extraction of the *Taraxacum* pollen into methanol for 24 h (protocol adapted from [3,4]). The methanol was then evaporated at 60°C under reduced pressure and the chemical extract was resuspended in an aqueous methanolic solution (50:50, v/v). Liquid-liquid partitioning was performed three times against heptane. The pooled heptane extracts were then washed two times with aqueous methanol (50:50, v/v) that was recovered. The aqueous methanolic extract was evaporated under reduced pressure and dissolved in water in order to undergo a last partitioning against chloroform. The chloroformic fraction, containing the purified lactones, was evaporated to dryness and dissolved in 100 mL of an aqueous ethanolic solution (50:50, v/v). The lactone extract was characterized by liquid chromatography coupled to a mass spectrometer).

For the on-line LC-MS analyses, a Waters Alliance 2695 liquid chromatography device was used. The HPLC device was coupled to the Waters Quattro Premier mass spectrometer and consisted of a vacuum degasser, a quaternary pump and an autosampler. Sample volumes of 15μl were injected. Chromatographic separation was performed on a non-polar column (Kinetex C18; 2.1 Å~ 100 mm; 2.6 μm; Phenomenex) at 40°C. The mobile phase was programmed with a constant flow (0.2 ml/min) of 60% of eluant A (water HPLC, 0.1% formic acid) and 40% of eluant B (acetonitrile) during 15 minutes. The ESI conditions were: capillary voltage 3.1 kV; cone voltage 30V; source temperature 100 °C; desolvation temperature 300 °C. Dry nitrogen was used as the ESI gas with a flow rate of 50 l/h for the gas cone and 600 l/h for the desolvation gas. The single-stage ESI-MS spectra were recorded by scanning the first quadrupole analyzer between m/z 250 and 1600. For the ESI-MS-MS experiments, the ions of interest were mass-selected by the first quadrupole. The selected ions were then submitted to collision against argon in the hexapole collision cell (pressure estimated at 0.9–1 mbar). The product ions were finally mass measured with the second quadrupole analyzer.

Major peaks were detected at *m/z* 427 and *m/z* 415. They correspond to sesquiterpene lactones previously reporeted in *Taraxacum* roots, namely Ixerin D and 13-dihydroacid taraxinic β-pyranosyl ester (*m/z* 415) (Fig. 2) [5]. Another peak at *m/z* 281 was hypothetically identified as 1β,10α,4α,5β-diepoxy-6β-hydroxy- glechoman-8α,12-olide, a sesquiterpene lactone also reported in *Smyrnium* genus (Apiaceae) (Fig. 2) [6].


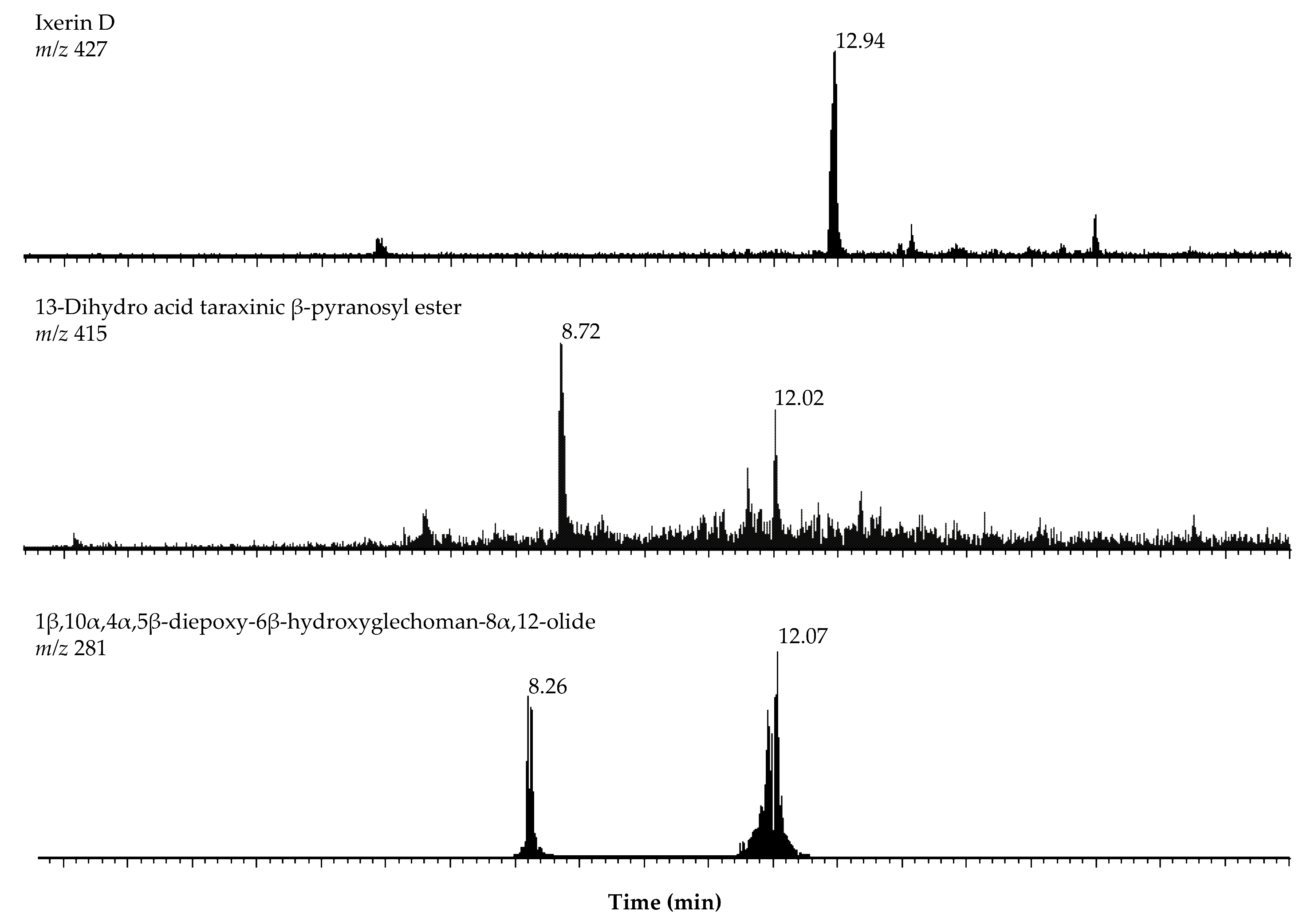


**Figure 2.** LC-MS extracted ion chromatograms of the major sesquiterpene lactones detected in *Taraxacum* pollen loads.

References

1. Vanderplanck, M. ; Michez, D. ; Vancraenenbroeck, S. ; Lognay, G. Micro-quantitative method for analysis of sterol levels in honeybees and their pollen loads. *Anal. Lett.* **2011**, *44*, 1807-1820.
2. Vanderplanck, M. ; Declèves, S. ; Roger, N. ; Decroo, C. ; Caulier, G. ; Glauser, G. ; Gerbaux, P. ; Lognay, G. ; Richel, A. ; Escaravage, N. ; Michez, D. Is non-host pollen suitable for generalist bumblebees ? *Insect Sci.* **2018**, *25(2)*, 259-272.
3. Mustakerova, E. ; Todorova, M. ; Tsankova, E. Sesquiterpene lactones from *AChillea collina* Becker. *Z. Naturforsch. C* **2002**, *57*, 568-570.
4. Basarkar, U.G. ; Saoji, A.A. Isolation, characterization of sesquiterpene parthenin and its estimation from Parthenium hysterophorus pollen. IJETCAS 2013, 5(4), 364-368.
5. Kiesel, W. ; Barszcz, B. Further sesquiterpenoids and phenolics from *Taraxacum officinale*. *Fitoterapia* **2000**; *71*, 269-273.
6. El-Gamal A.A. Sesquiterpene lactones from *Smyrnium olusatrum*. *Phytochem.* **2001**, *57*, 1197-1200.

**Table S1 – Effects of diet treatments on *B. terrestris* in micro-colonies**, mean (SE) (n = 10 for each treatment). Different letters indicate significant differences (*p* < 0.01) between two diet treatments (post-hoc Tukey analyses with FDR adjustment).

| **Parameters** | **Treatment** | | | | | **Statistics^1^** |
| --- | --- | --- | --- | --- | --- | --- |
|  | **Control** | ***Taraxacum*** | **Crushed**  ***Taraxacum*** | **Phytosterol** | **Lactone** |  |
| Pollen collection (g) | 17.29 (0.86) ^a^ | 9.11 (1.23) ^b^ | 9.19 (1.11) ^b^ | 17.59 (0.91) ^a^ | 17.00 (0.78) ^a^ | χ^2^ = 88.04, df = 4, ***p* < 0.001** |
| Syrup collection (g) | 73.68 (1.75) ^ab^ | 64.51 (4.94) ^bc^ | 62.21 (3.68) ^c^ | 78.09 (3.35) ^a^ | 69.14 (2.28) ^abc^ | χ^2^ = 18.73, df = 4, ***p* < 0.001** |
| Pollen dilution | 4.33 (0.15) ^b^ | 7.73 (0.62) ^a^ | 7.43 (0.68) ^a^ | 4.48 (0.17) ^b^ | 4.12 (0.16) ^b^ | χ^2^ = 95.52, df = 4, ***p* < 0.001** |
| Mass of total eclosed offspring (g) | 9.29 (0.54) ^a^ | 2.70 (0.72) ^b^ | 3.20 (0.67) ^b^ | 7.86 (0.62) ^a^ | 8.83 (0.56) ^a^ | χ^2^ = 122.41, df = 4, ***p* < 0.001** |
| Pollen efficiency | 0.54 (0.01) ^a^ | 0.26 (0.04) ^b^ | 0.31 (0.04) ^b^ | 0.44 (0.02) ^a^ | 0.52 (0.02) ^a^ | χ^2^ = 100.54, df = 4, ***p* < 0.001** |
| Larval ejection | 0.06 (0.02) ^c^ | 0.24 (0.05) ^a^ | 0.08 (0.02) ^bc^ | 0.15 (0.03) ^ab^ | 0.10 (0.02) ^abc^ | χ^2^ = 21.14, df = 4, ***p* < 0.001** |
| Number of eggs | 14 (3) | 8 (2) | 4 (3) | 16 (6) | 16 (7) | χ^2^ = 8.10, df = 4, *p* = 0.088 |
| Number of non-isolated larvae | 48 (7) ^a^ | 18 (4) ^b^ | 30 (6) ^ab^ | 30 (6) ^ab^ | 41 (10) ^ab^ | χ^2^ = 15.54, df = 4, ***p* = 0.004** |
| Number of pre-defecating larvae | 9 (2) | 4 (2) | 5 (1) | 5 (2) | 9 (2) | χ^2^ = 9.82, df = 4, *p* = 0.043 |
| Number of post-defecating larvae | 2 (1) | 1 (0) | 1 (0) | 3 (1) | 2 (1) | χ^2^ = 11.79, df = 4, *p* = 0.019 |
| Number of pupae | 11 (1) ^a^ | 3 (1) ^c^ | 4 (1) ^bc^ | 9 (1) ^a^ | 8 (2) ^ab^ | χ^2^ = 32.50, df = 4, ***p* < 0.001** |
| Number of non-emerged drones | 1 (0) | 0 (0) | 0 (0) | 1 (0) | 1 (0) | χ^2^ = 8.79, df = 4, *p* = 0.067 |
| Number of emerged drones | 9 (1) ^a^ | 1 (1) ^b^ | 2 (1) ^b^ | 9 (1) ^a^ | 10 (1) ^a^ | χ^2^ = 49.88, df = 4, ***p* < 0.001** |

χ^2^: value of the type II Wald chi square; df: degree of freedom; *p*: p-value of the type II Wald chi square. Significant results are depicted in bold (*p* < 0.01).

**Figure S1 – Pollen collection by *B. terrestris* in micro-colonies over time.** The thickness of the horizontal line represents the collection values for a single micro-colony over time.

| 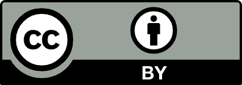 | © 2020 by the authors. Submitted for possible open access publication under the terms and conditions of the Creative Commons Attribution (CC BY) license (http://creativecommons.org/licenses/by/4.0/). |
| --- | --- |
